# Neobavaisoflavone Ameliorates Memory Deficits and Brain Damage in Aβ_25‐35_‐Induced Mice by Regulating SIRT1

**DOI:** 10.1111/cns.70068

**Published:** 2024-10-11

**Authors:** Fengxiao Hao, Mengnan Zeng, Bing Cao, Xiwen Liang, Kaili Ye, Xinmian Jiao, Weisheng Feng, Xiaoke Zheng

**Affiliations:** ^1^ College of Pharmacy Henan University of Chinese Medicine Zhengzhou China; ^2^ The Engineering and Technology Center for Chinese Medicine Development of Henan Province Zhengzhou China; ^3^ Co‐construction Collaborative Innovation Center for Chinese Medicine and Respiratory Diseases by Henan & Education Ministry of P.R Zhengzhou China

**Keywords:** Alzheimer's disease, brain injury, neobavaisoflavone, neuroinflammation, oxidative stress, SIRT1

## Abstract

**Background:**

Alzheimer's disease (AD) is a common chronic neurodegenerative disease in older people, and there is no specific treatment that can stop or reverse its progression. Neobavaisoflavone (NBIF) is a flavonoid that has been shown to have neuroprotective effects, but its role in AD has not been revealed. The present study investigated the role and mechanism of NBIF on Aβ_25‐35_‐induced brain injury.

**Methods:**

In this experiment, the AD mouse model was established by injection of Aβ_25‐35_ peptides (200 μM, icv), and Donepezil (Don, 10 mg/kg/days), NBIF‐L (15 mg/kg/days), and NBIF‐H (30 mg/kg/days) were administered orally for 4 weeks. Learning memory, hippocampal pathological changes, pathological markers, apoptosis, oxidative stress, inflammation, immune cells were measured in mice. Network pharmacology combined with the GEO database led to the identification of SIRT1, a key target for NBIF intervention in AD, and levels of SIRT1, p‐STAT3 and FOXO1 were measured. In addition, the antagonistic activity of SIRT1 transfection silencing against NBIF in Aβ_25‐35_‐induced in N9 cells and N2a‐APP69 cells was investigated to assess whether the effects caused by NBIF were mediated by SIRT1.

**Results:**

The results showed that NBIF ameliorated learning memory and hippocampal neuronal damage, reduced pathological markers, apoptosis, oxidative stress and neuroinflammation, and modulated immune cells. SIRT1 is a key target for NBIF intervention in AD, and NBIF upregulates SIRT1 and reduces the expression levels of p‐STAT3 and FOXO1. Furthermore, silencing SIRT1 effectively reduced the protective effect of NBIF on Aβ_25‐35_‐induced N9 cells and N2a‐APP69 cells, which indicated that the protective effect of NBIF on AD is related to SIRT1.

**Conclusions:**

NBIF ameliorated Aβ_25‐35_‐induced brain injury by inhibiting apoptosis, oxidative stress, and neuroinflammation, which may be mediated through SIRT1 signaling. These findings provide a rationale for NBIF in the treatment of AD and help facilitate the development of clinical therapeutic agents for AD.

## Graphical abstract

NBIF ameliorates Aβ_25‐35_‐induced brain injury by inhibiting apoptosis, oxidative stress and neuroinflammation through the SIRT1‐mediated STAT3/FOXO1 signaling pathway.

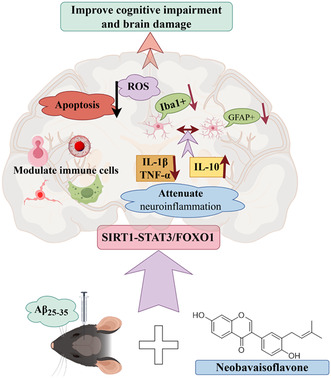

**Abbreviations**
Aβ_1‐40_
: beta amyloid beta 1‐40
Aβ_1‐42_
: beta amyloid beta 1‐42
AD: Alzheimer's disease
ADAM10: a disintegrin and metalloprotease 10
APP: β‐amyloid precursor protein
BACE1: beta‐site APP cleaving enzyme 1
Bax: associated X protein
Bcl‐2: B‐cell lymphoma 2
Caspase‐3: cysteine‐dependent aspartate‐specific proteases 3
Caspase‐9: cysteine‐dependent aspartate‐specific proteases 9
Don: donepezil
FOXO1: forkhead box protein O1
GAPDH: glyceraldehyde‐3‐phosphate
GSH‐Px: glutathione peroxidase
HE: hematoxylin–eosin
IL‐1β: interleukin 1 beta
IL‐10: interleukin 10
MDA: malondialdehyde
MTT: methyl thiazolyl tetrazolium
MWM: morris water maze
NBIF: neobavaisoflavone
NOR: novel object recognition test
OFT: open field test
p‐Tau: phosphorylated Tau protein
ROS: reactive oxygen species
SIRT1: sirtuin 1
SOD: superoxide dismutase
STAT3: signal transducer and activator of transcription 3
TNF‐α: tumor necrosis factor alpha

## 1 Introduction

Alzheimer's disease (AD) is one of the most common neurodegenerative diseases occurring in old age characterized by memory loss and cognitive impairment [1]. Report shows AD has become the fifth leading cause of death besides cerebrovascular disease, ischemic heart disease, chronic obstructive pulmonary disease, and lung cancer [2]. The Seventh National Population Census of China in 2020 found that the number of people in China over the age of 60 accounted for 32.2% of the country's total population [3]. With the aging of the population, the prevalence of AD is also on the rise every year, increasing the burden on families and society.

Although plaques formed by extracellular Aβ deposits and neurofibrillary tangles formed by intracellular tau proteins are considered to be the main pathological features of AD [4], the specific pathogenesis is complex and has not been fully clarified. Research has been pointed out that excessive deposition of Aβ in the development of AD also affects the abnormal activation of glial cells, leading to the release of reactive oxygen species and pro‐inflammatory cytokines, which promotes neuroinflammation and neuronal apoptosis, thereby exacerbating brain damage [5]. Although there are medications to relieve the symptoms of AD, they do not stop the disease from progressing [6]. Therefore, it is important to look for drugs to treat AD.

Neobavaisoflavone (NBIF) is a flavonoid that has a variety of pharmacological uses including antibacterial [7], anti‐inflammatory [8], and antitumor [9]. It has been reported that NBIF, as an isoprenoid isoflavone, is more accessible to the brain and is almost uniformly distributed in the nucleus accumbens [10]. In addition, NBIF can ameliorate age‐related diseases [11], inhibit LPS‐induced NO production in BV‐2 cells and H_2_O_2_‐induced HT22 cell damage [12], and have neuroprotective effects. Nevertheless, it is still unclear whether NBIF exerts a neuroprotective effect in the brain injury of AD. Aβ_25‐35_, as the shortest segment that retains the toxicity of full‐length Aβ (1–40/42), has been shown to be useful for constructing AD models [13]. Our group has also used Aβ_25‐35_ to construct an AD model in the previous period and demonstrated the effects and mechanism of adenosine, amentoflavone, *Corallodiscus flabellata* B. L. Burtt extract and isonuomioside A to intervene in AD [14, 15, 16]. In the present study, we used Aβ_25‐35_ to construct an AD model and chose donepezil, the most commonly used drug in the clinical treatment of AD [17], as a positive control aiming to investigate the effect of NBIF on AD brain injury and to explore its potential mechanisms for AD treatment.

## 2 Materials and Methods

### 2.1 Medicine

Aβ_25‐35_ peptide was obtained from Sangon Biotech Co., Ltd. (Shanghai, China), dissolved in double‐distilled water (1 mM) and aggregated by incubating at 37°C for 7 days. Donepezil (CAS: 110119‐84‐1, purity ≥ 98%) was obtained from Shanghai Yuanye Biotechnology Co., Ltd. NBIF (CAS: 41060‐15‐5, purity ≥ 99%) was purchased from Taoshu (Shanghai, China).

### 2.2 Animals

**FIGURE 1:**
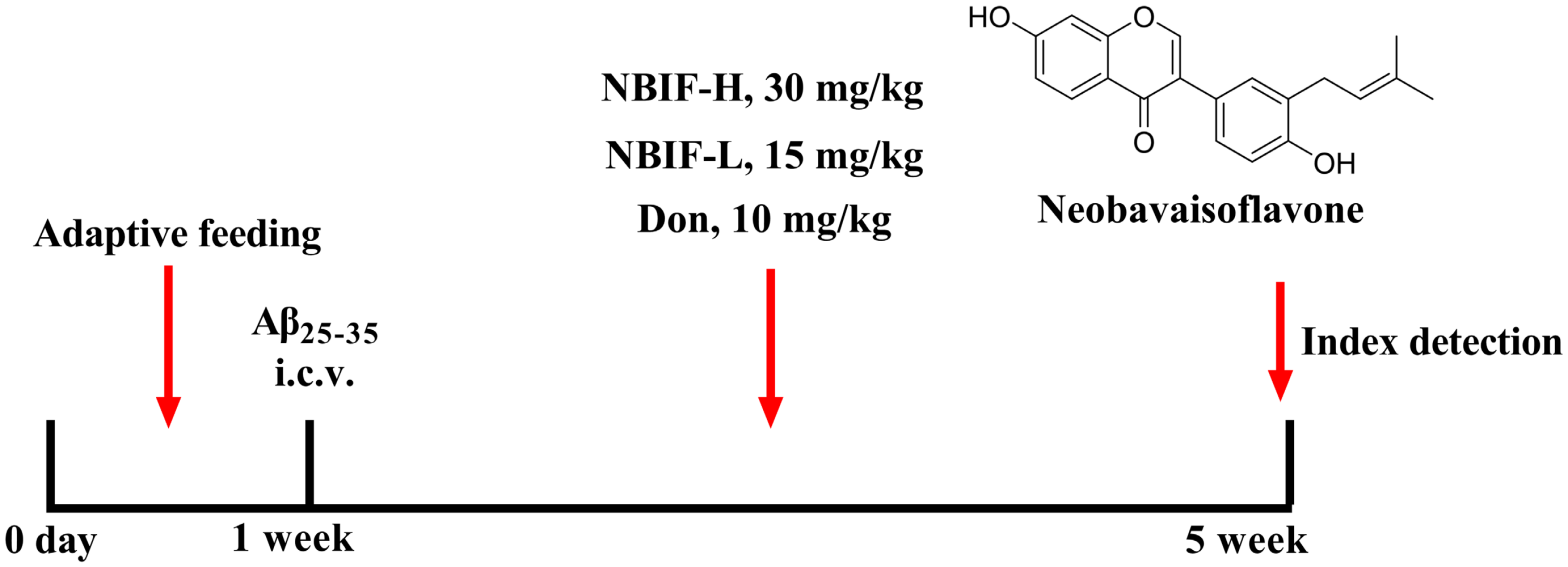
Process of experiment.

Male C57BL/6J mice (6–7 weeks old, *n* = 50) was obtained from Beijing Huafukang Biotechnology Co. Ltd. (protocol number: SCXK [Jing] 2019‐0008) and kept in a clean‐grade animal room at 18°C–24°C, alternating day and night, with free access to water and food. After 7 days of adaptive feeding, mice were injected intracerebroventricularly (icv) as previously described [13]. Forty mice were randomly selected as the AD model group to be injected with Aβ_25–35_, and the remaining ten mice were injected with an equal amount of saline as the sham operation group (Sham). After 3 days of recovery, mice in the AD model group were randomly divided into four groups: model (M), donepezil‐treated (10 mg/kg, Don), low‐dose NBIF‐treated (15 mg/kg, NBIF‐L), and high‐dose NBIF‐treated (30 mg/kg, NBIF‐H). Afterwards, the drug was administered by gavage for 28 days, and distilled water was gavaged in the groups of sham and M. All behavioral tests were completed prior to animal necropsy. The experimental procedure is shown in Figure 1. All procedures were performed according to the Guidelines for Care and Use of Laboratory Animals of the Henan University of Chinese Medicine, and experiments were approved by the Animal Ethics Committee of the Henan University of Chinese Medicine (Approval number: DWLL2018080003).

### 2.3 Behavioral Tests

#### 2.3.1 Open Field Test (OFT)

As previously reported [18], the OFT‐100 analysis system (Taming, Chengdu, China) was used to record the movement distance of mice over a 5‐min period.

#### 2.3.2 Novel Object Recognition Test (NOR)

The mice's exploration time for new and old objects was recorded within 5 min, and the preference index and discrimination index were calculated according to our previously reported methods [19].

#### 2.3.3 Morris Water Maze Test (MWM)

MWM was used to assess the spatial learning and memory cognitive abilities of animals [20]. On the first day of the experiment, the mice were allowed to explore freely for 5 min and were guided to reach the platform. After that, the Animal Behavioral Trajectory Video Analysis System V3.0 (Labmaze, Beijing Zhongshi Ditron Technology Development Co., Ltd.) was used to record the time of mice reached the platform on Days 2–6. Removed the platform on Day 7, and recorded the residence time of the mice in the original quadrant of the platform within 60 s.

### 2.4 Histopathology of Brain Tissues

Brain tissues were fixed in 4% paraformaldehyde 24 h and embedded in paraffin. Specimens were cut into 5 μm sections and then stained with hematoxylin and eosin (H&E) or cresyl violet (Nissl stain). We observed the morphological changes in hippocampal neurons under an optical microscope (400×). The number of cells in the hippocampus was quantitated using the Image‐Pro Plus software.

### 2.5 Immunofluorescence Staining

Brain tissue sections were first deparaffinized and antigenically repaired, and endogenous peroxidase was blocked with 3% hydrogen peroxide. Serum was added to close the non‐specific sites and incubated for 30 min at room temperature, and primary antibodies (GFAP [1:100, GB11096, Servicebio], Iba1 [1:100, GB113502, Servicebio]) were added immediately and incubated at 4°C overnight. After washing, add secondary antibody and incubate at room temperature for 30 min, and finally seal the film with neutral resin. Fluorescence images were scanned and obtained by Wuhan Servicebio Biotechnology Co., Ltd., screenshots were taken using CaseViewer software, and fluorescence intensity was quantified by ImageJ software.

### 2.6 Biochemical Indicators

Preparation of 10% brain tissue homogenates to detect the levels of mouse beta amyloid beta 1–42 (Aβ_1‐42_, MM‐0220M1, Meimian, Jiangsu, China), mouse beta amyloid beta 1–40 (Aβ_1‐40_, MM‐0461M1, Meimian, Jiangsu, China), phosphorylated Tau protein (p‐Tau, MM‐45352M1, Meimian, Jiangsu, China); total superoxide dismutase (T‐SOD, A001‐3‐2), glutathione peroxidase (GSH‐Px, A005‐1‐2), malondialdehyde (MDA, A003‐1‐2) kits were purchased from Nanjing Jiancheng Bioengineering Institute (Nanjing, China). The levels of mousetumor necrosis factor‐α (TNF‐α), mouse interleukin‐10 (IL‐10), mouse interleukin‐1β (IL‐1β) were detected by ABplex Mouse 4‐Plex Custom Panel (RK04381, ABclonal).

### 2.7 Flow Sight and Flow Cytometry Analysis of Apoptosis, Reactive Oxygen Species (ROS) and Mitochondrial Membrane Potential (MMP) Positive Rate

Mouse primary hippocampal cells were obtained as described previously; N9 cells were treated with drugs for 24 h and then centrifuged to obtain single cells. The primary brain cells and N9 cells were then treated according to the instructions of the PE Annexin V apoptosis assay kit (BD Biosciences, 559763), the ROS assay kit (Solarbio, CA1410), and MMP assay kit with JC‐1 (Solarbio, M8650), respectively. The apoptosis and ROS levels in primary brain cells were measured and analyzed by Flowsight (Luminex, USA), while JC‐1 was measured using flow cytometry (BD FACSAria III, USA).

### 2.8 Detection of Immune Cells in the Blood of Aβ_25‐35_‐Injected Mice

The mice blood cells were divided into 4 portions with 100 μL of cells per portion. Regulatory T (Treg) cells were detected using anti‐CD4 (11‐0041‐85, Invitrogen, New York, USA), anti‐CD25 (12‐0251‐81, Invitrogen, New York, USA), and anti‐FOXP3 (17‐5773‐82, Invitrogen, New York, USA) antibodies. The helper (Th) and cytotoxic (Tc) cells were detected by antibody‐CD8a (11‐0081‐85, Invitrogen, New York, USA), antibody‐CD4 (12‐0041‐82, Invitrogen, New York, USA), and antibody‐CD3e (17‐0031‐82, Invitrogen, New York, USA). Natural killer (NK) cells were detected using anti‐CD3e (17‐0031‐82, Invitrogen, New York, USA) and anti‐NK1.1 (12‐5941‐82, Invitrogen, New York, USA) antibodies. Myeloid‐derived suppressor cells (MDSCs) were detected using anti‐CD11b (17‐0112‐81, Invitrogen, New York, USA) and anti‐Ly‐6G (12‐9668‐82, Invitrogen, New York, USA) antibodies. The cellular fluorescence intensity was analyzed by BD FACSAria III flow cytometry.

### 2.9 Western Blotting

Suitable quantities of mouse brain tissue were homogenized in lysis buffer, the supernatant was collected, the protein concentration was determined using a BCA Protein Extraction Kit (Solarbio, Beijing, China), and the on‐sample protein was produced using loading buffer. The samples were separated by SDS‐PAGE gel, after which they were transferred to PVDF membranes, which were combined with primary antibodies (BACE1 [5606S], ADAM10 [bs‐3574R], SIRT1 [bs‐0921R], p‐STAT3 [bs‐1658R], STAT3 [9139S], FOXO1 [bs‐9439R], Caspase‐3 [ab13847], Caspase‐9 [ab202068], Bax [ab32503], Bcl‐2 [ab59348], GAPDH [GB15002], β‐Tubulin [I0094‐I‐AP]) were incubated overnight at 4°C, followed by incubation with secondary antibodies protected from light for 1 h. Odyssey CLx infrared fluorescence scanning imaging system (Clx, LiCOR, MO, USA) was used to create and quantify the images.

### 2.10 Network Pharmacological Approach

PharmMapper (http://www.lilab‐ecust.cn/pharmmapper/) and Swiss Target Prediction (http://www.swisstargetprediction.ch/) were used to collect targets of NBIF. Drugbank (https://www.drugbank.ca/), GeneCards (https://genecards.org/), DisGeNET (https://www.disgenet.org/) and OMIM (https://www.omim.org/) were used to collect targets related to AD. STRING database was used to visualize protein–protein interaction (PPI) network. Metascape (https://metascape.org/) were used to perform KEGG enrichment analysis and gene ontology (GO) functional annotation.

### 2.11 GEO Information Acquisition

Relevant datasets were screened with the keywords AD and SIRT1 from GEO to obtain the GeneChip dataset GSE142633 (microarray platform GPL24247) eligible for the study. This dataset contains wild‐type mouse samples and 5 × FAD mouse samples. Download the relevant files and use the Lianchuan BioCloud platform to draw a pair of violin plots to visualize the changes in SIRT1 levels.

### 2.12 Molecular Docking

The module was employed to investigate the potential interactions between NBIF and SIRT1. The SIRT1 protein was used as the receptor (PDB: 4IF6), and NBIF was obtained from the website (https://pubchem.ncbi.nlm.nih.gov/). All docking simulations were performed using default parameters, and binding affinities were estimated by total scores. The docking process was performed by SailVina and The Protein‐Ligand Interaction Profiler website (https://plip‐tool.biotec.tu‐dresden.de/plip‐web/plip/index) was used to analyze the binding properties of the ligands to each protein.

### 2.13 Cell Culture and Treatment

#### 2.13.1 N9 Microglial Cells

N9 microglial cells (purchased from Beina Biotechnology Co., Ltd.) were maintained in RPMI‐1640 medium (Gibco, 31800‐022, USA) with 10% fetal bovine serum (FBS, CM1002L, CellCook, China) in a 37°C, 5% CO_2_ incubator. In in vitro experiments, N9 microglia cells were inoculated into 96‐well plates at a density of 3 × 10^4^ cells/well to screen cell viability and into 6‐well plates at a density of 6 × 10^4^ cells/well for other experiments.

#### 2.13.2 Mouse Neuroblastoma N2a Cells (N2a) and N2a‐APP695 Cells

N2a cells purchased from Wuhan Pricella Biotechnology Co., Ltd. N2a‐APP695 stably transfected cells were obtained by overexpression of APP695 from N2a cells, which was done by Hanheng Biotechnology (Shanghai) Co., Ltd. N2a and N2a‐APP695 cells were maintained in Dulbecco's modified Eagle's medium (DMEM) (Gibco, 12100‐061, USA) with 10% fetal bovine serum (FBS, CM1002L, CellCook, China) in a 37°C, 5% CO_2_ incubator. In in vitro experiments, N2a‐APP695 cells were inoculated into 96‐well plates at a density of 3 × 10^4^ cells/well to screen cell viability and detect pathway‐associated protein expression, and into 6‐well plates at a density of 6 × 10^4^ cells/well for other experiments.

#### 2.13.3 SIRT1 Silencing by Transfection

The cells were divided into the CON group, the Aβ_25‐35_ group (0.1 μM), and the NBIF group (the treatment for the NBIF group was (+) or (−) SIRT1 siRNA). When the cell density was approximately 60%, transfection was performed to silence SIRT1 in strict accordance with the instructions of GP‐transfect‐Mate (G04008, GenePharma, China). Replace all media after 4–6 h for modeling and drug administration. After 24 h, the total RNA extraction kit (R1200, Solarbio, China) was used to extract the RNA from each group, the SweScript RT I First Strand cDNA Synthesis Kit (G3330‐100, Servicebio, China) was used to reverse transcribe the sample into cDNA, and the 2 × Universal SYBR Green qPCR Master Mix (G3326‐15, Servicebio, China) was used to detect the mRNA levels of Sirt1 (TTCAGAACCACCAAAGCGGA, TCCCACAGGAGACAGAAACCC) and Gapdh (CCTCGTCCCGTAGACAAAATG, TGAGGTCAATGAAGGGGTCGT).

#### 2.13.4 Effect of NBIF on Aβ_25‐35_‐Induced N9 Microglial Cells Damage

N9 cells were treated with Aβ_25‐35_ (0.1 μM) and different concentrations of NBIF (0.1, 0.5, 1, 2, 5, 10, 20, and 30 μM), and cell viability was determined after 24 h using methylthiazolyldiphenyl‐tetrazolium bromide (MTT) assay at 490 nm. For experiments with (+) or (−) SIRT1 siRNA, 24 h after administration, cells were collected and assayed for apoptosis, ROS and JC‐1 levels using flow cytometry.

#### 2.13.5 Effect of NBIF on Pathway‐Related Protein Expression in N2a‐APP695

The level of APP protein expression in N2a versus N2a‐APP695 cells were detected using in cell western (ICW) to identify N2a‐APP695 cells as stably transient cells. Next, N2a‐APP695 cells were treated with different concentrations of NBIF (0.1, 0.5, 1, 2, 5, 10, 20, and 30 μM), and cell viability was determined by MTT at 490 nm after 24 h. In experiments using (+) or (−) SIRT1 siRNA, pathway‐associated protein levels were measured using ICW 48 h after administration.

### 2.14 In Cell Western (ICW)

N2a and N2a‐APP695 cells were fixed in paraformaldehyde for 15 min and permeabilized with Triton X‐100. Cells were then blocked with 5% BSA for 1.5 h; primary antibodies APP (ab32136), SIRT1 (bs‐0921R), p‐STAT3 (bs‐1658R), STAT3 (9139S), FOXO1 (bs‐9439R), GAPDH (GB15002) were added and incubated overnight at 4°C; fluorescently labeled secondary antibodies were added and incubated for 1 h and washed for imaging and analysis with Odyssey instruments (Clx, LiCOR, MO, USA).

### 2.15 Statistical Analysis

Data were analyzed using IBM SPSS 26.0 software and expressed as the mean ± standard deviation (SD). All data were checked for normal/Gaussian distribution using the Shapiro–Wilk test. Paired‐samples *t*‐test (two groups) and one‐way analysis of variance (ANOVA) followed by the least significant difference test (LSD) were used to compare differences between groups. Data that do not exhibit a normal/Gaussian distribution were analyzed via a non‐parametric equivalent. Probability values < 0.05 were considered statistically significant.

## 3 Results

### 3.1 NBIF Ameliorated Cognitive Deficits and Learning Memory in Aβ_25‐35_ Induced Mice

**FIGURE 2:**
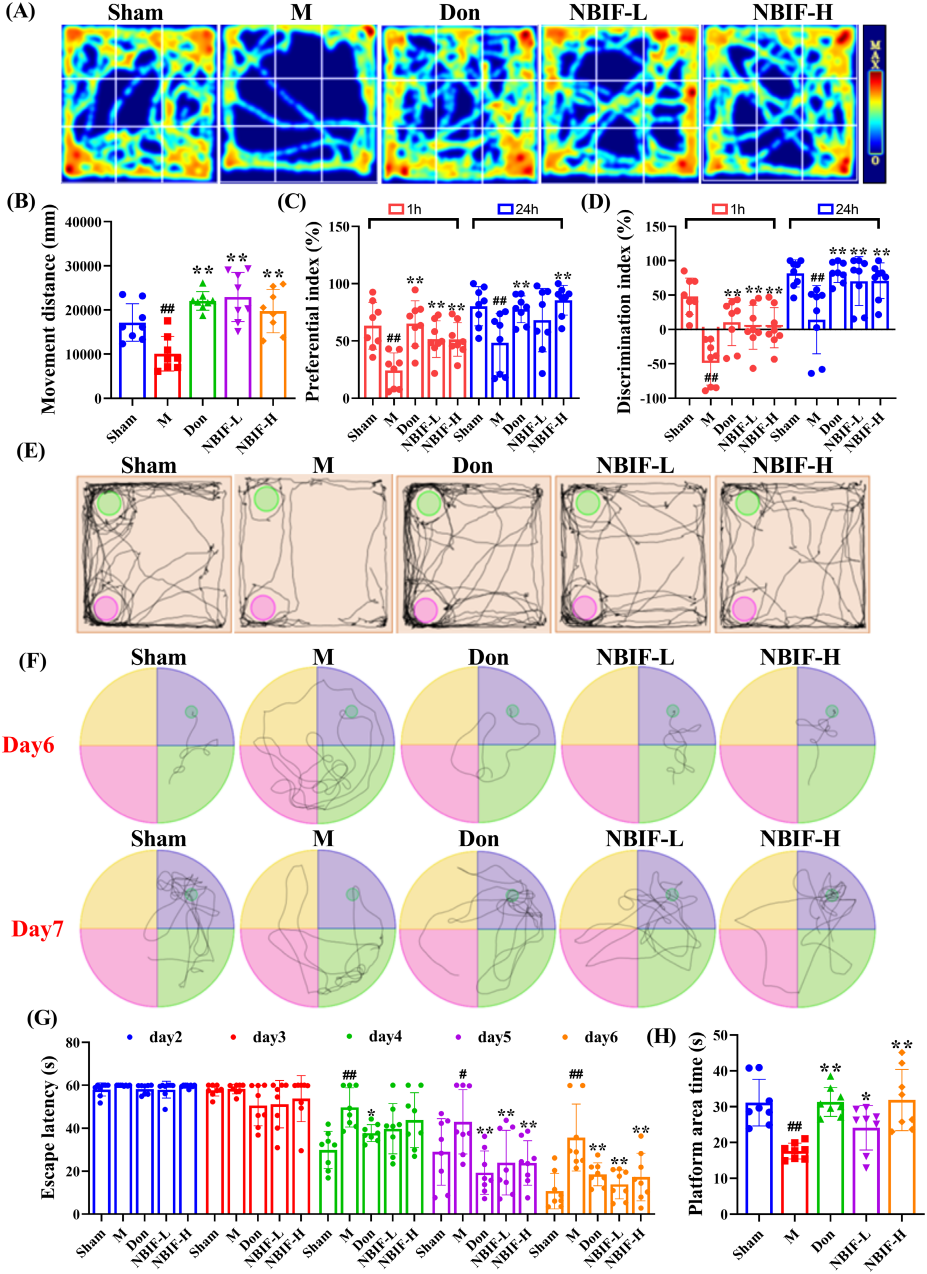
NBIF ameliorated cognitive deficits and learning memory in Aβ25‐35 induced mice. (A) Representative heatmaps of OFT. (B) Quantified results of OFT. (C and D) Quantitative results of preference index and discrimination index. (E) Representative images of NOR (green color represents new object, red color represents old object). (F) Representative images of MWM. (G and H) Quantification of mice escape latency at 2–6 days and movement time in the platform area after platform removal. Data were expressed as the mean ± SD (*n* = 8). ^#^ *p* < 0.05 and ^##^ *p* < 0.01 versus Sham; *p < 0.05 and **p < 0.01 versus M.

To assess the effect of NBIF on cognitive deficits and learning memory in Aβ_25‐35_‐injected mice, we performed OFT, NOR, and MWM experiments. The OFT results showed that the movement distance of the Aβ_25‐35_‐induced mice was significantly lower than mice in the Sham group (*p* < 0.01), and could be increased by NBIF (*p* < 0.01, Figure 2A,B). The NOR test showed that the preference and discrimination index of the new object was significantly reduced in the M group mice (*p* < 0.01), while the administration of NBIF significantly improved these indexes (*p* < 0.01, Figure 2C–E). MWM was used to further assess learning memory capacity, and it showed that the time to find the plateau was significantly shorter in all groups of mice from Days 4–6. After removed the platform, mice in the Don, NBIF‐L, and NBIF‐H groups showed different increases in the time spent in the platform area (*p* < 0.05 or *p* < 0.01, Figure 2F–H). From these results, it can be observed that NBIF ameliorated cognitive deficits and learning memory in Aβ_25–35_‐induced AD mice similarly to the positive control Don.

### 3.2 NBIF Alleviated Neuronal Damage in the Hippocampus of Aβ_25‐35_‐Induced Mice

**FIGURE 3:**
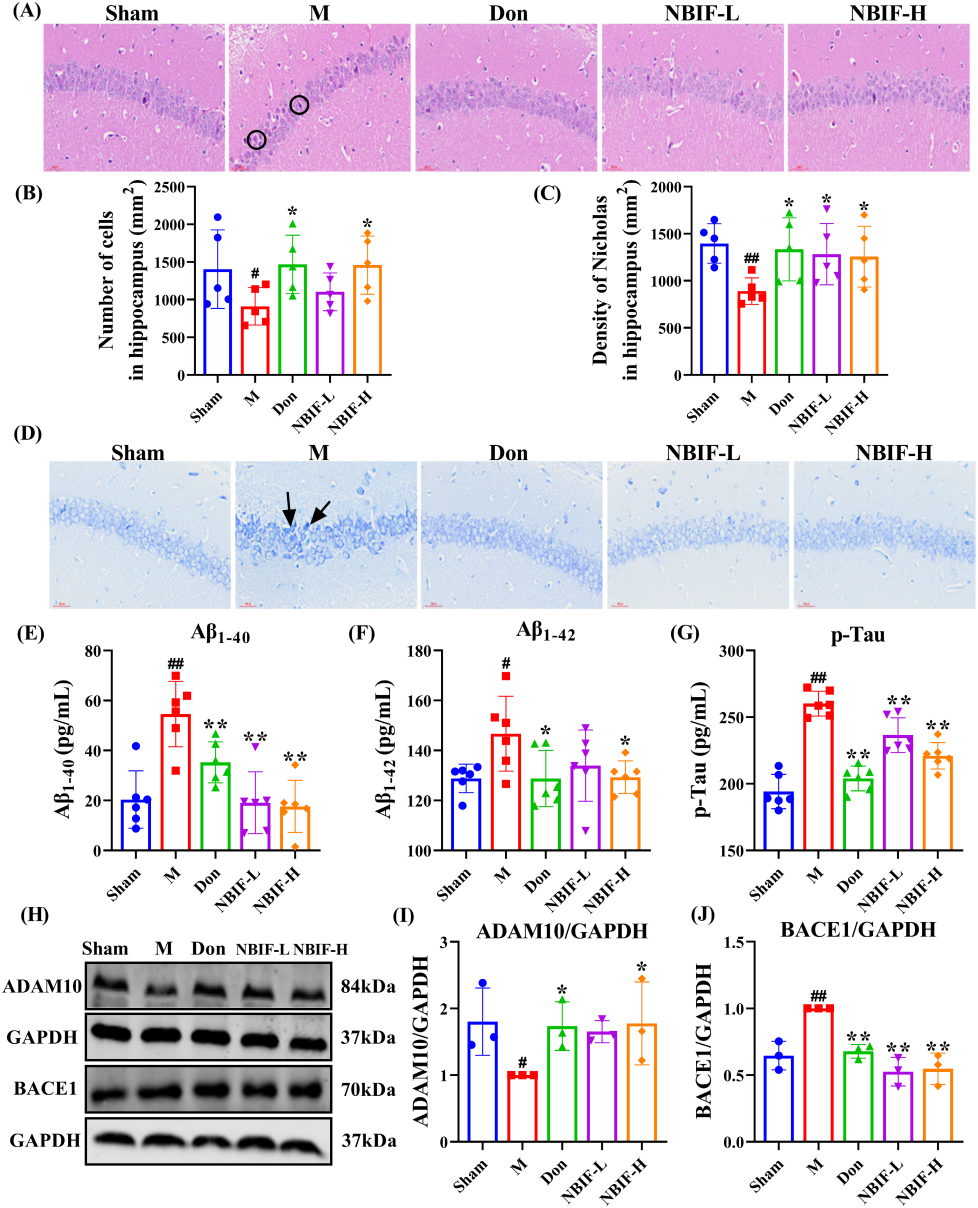
NBIF alleviated neuronal damage in the hippocampus of Aβ_25‐35_‐induced mice. (A and B) Characteristic picture and quantitative analysis of HE staining (×400, *n* = 5, scale bar = 30 μm). (C and D) Quantitative results and representative images of Nissl staining (×400, *n* = 5, scale bar = 30 μm). (E–G) Analysis of Aβ_1‐40_, Aβ_1‐42_, and p‐Tau in mice brain tissue (*n* = 6). (H–J) Representative WB images and quantitative analysis of the protein expressions of ADAM10 and BACE1 (*n* = 3). Data were expressed as the mean ± SD. ^#^ *p* < 0.05 and ^##^ *p* < 0.01 versus Sham; *p < 0.05 and **p < 0.01 versus M.

The hematoxylin–eosin (HE) and Nissl stain results indicated that hippocampal cells in the M group underwent crumpling and deepening of staining, and the number of normal neuronal cells was significantly reduced (Figure 3A–D, black circle and arrow). In contrast, NBIF attenuated hippocampal neuronal damage variously (*p* < 0.05). Meanwhile, we found that the levels of Aβ_1‐40_, Aβ_1‐42_, and p‐Tau in Aβ_25‐35_‐induced mice were higher than the sham group, and these were significantly reduced by administration of NBIF (*p* < 0.01 or *p* < 0.05, Figure 3E–G). Since Aβ production is closely linked to APP hydrolysis, we examined the expression of key enzymes in the APP hydrolysis process and found that NBIF increased ADAM10 levels and inhibited BACE1 expression (*p* < 0.01 or *p* < 0.05, Figure 3H–J). These findings showed that both Don and NBIF attenuated hippocampal neuronal damage and reduced the expression levels of pathological markers Aβ_1‐40_, Aβ_1‐42_, and p‐Tau, possibly by affecting APP hydrolysis, with Don and NBIF‐H showing better improvement.

### 3.3 NBIF Reduced Apoptosis, Oxidative Stress, and MMP in Aβ_25‐35_‐Induced Mice

**FIGURE 4:**
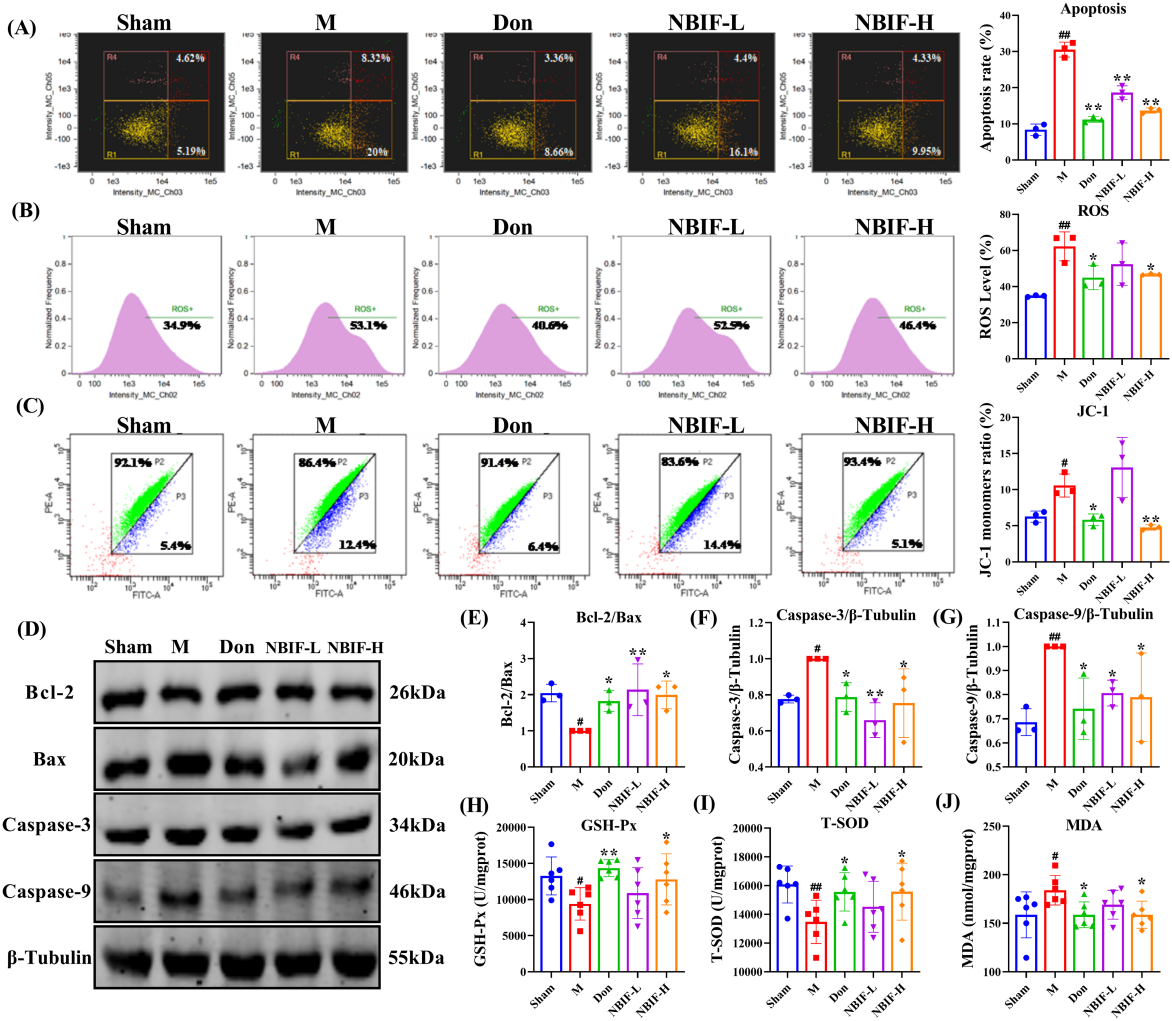
NBIF reduced apoptosis, oxidative stress, and MMP in Aβ_25‐35_‐induced mice. (A–C) Representative images and quantitative results of apoptosis, ROS, and JC‐1 monomer. (D–G) Representative WB images and quantitative analysis of the protein expressions of Bcl‐2/Bax, Caspase‐3, and Caspase‐9. (H–J) Analysis of GSH‐Px, T‐SOD and MDA in mice brain tissue (*n* = 6). Data were expressed as the mean ± SD (*n* = 3 or 6). ^#^ *p* < 0.05 and ^##^ *p* < 0.01 versus Sham; *p < 0.05 and **p < 0.01 versus M.

We assessed apoptosis, oxidative stress and MMP levels in brain tissue to investigate whether NBIF affects brain damage in Aβ_25‐35_‐induced mice. The results of flow cytometry analysis showed that NBIF reversed the increase of apoptosis, ROS and JC‐1 monomer in brain tissue of Aβ_25‐35_‐induced mice (*p* < 0.01 or *p* < 0.05, Figure 4A–C). Furthermore, we found that NBIF also upregulated the levels of Bcl‐2/Bax, T‐SOD and GSH‐Px (*p* < 0.01 or *p* < 0.05, Figure 4E,H,I), and decreased the levels of Caspase‐3, Caspase‐9, and MDA (*p* < 0.01 or *p* < 0.05, Figure 4F,G,J). The above results demonstrate that Don and NBIF reduced apoptosis and oxidative stress in Aβ_25‐35_‐induced mice. Among them, Don and NBIF‐H was more effective than NBIF‐L.

### 3.4 NBIF Modulated Immune Cells in Aβ_25‐35_‐Induced Mice

**FIGURE 5:**
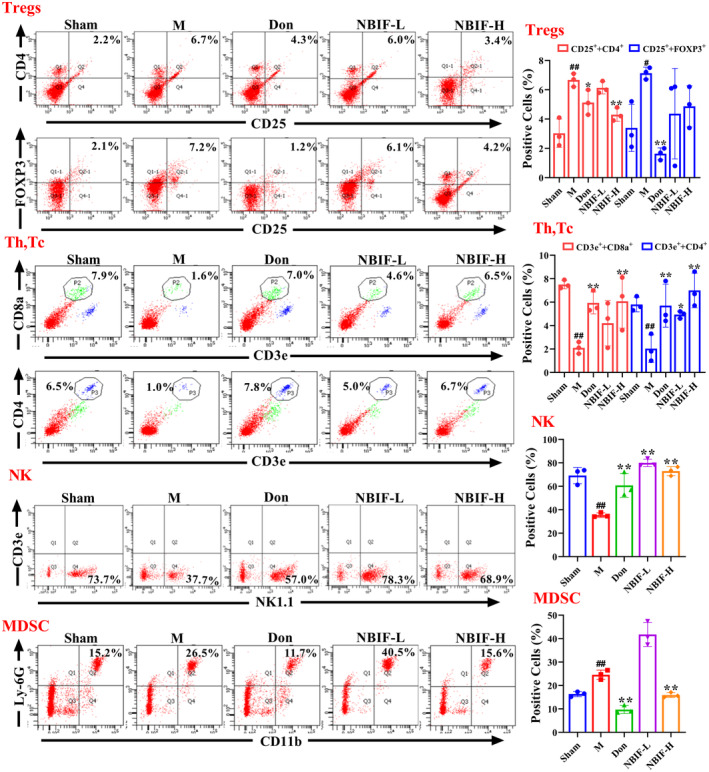
NBIF regulates the proportion of immune cells in the blood of Aβ_25‐35_‐induced mice. Representative images and quantitative results of Tregs, Th, Tc, NK, MDSC cells. Data were expressed as the mean ± SD (*n* = 3). ^#^ *p* < 0.05 and ^##^ *p* < 0.01 versus Sham; *p < 0.05 and **p < 0.01 versus M.

The immune cell levels in the blood may reflect the damage to the organism to some extent. Therefore, we examined the levels of immune cells in blood and found that NBIF significantly increased the Th, Tc, and NK cells, and decreased the percentage of MDSCs and Tregs cells in Aβ_25‐35_‐induced mice (*p* < 0.01 or *p* < 0.05, Figure 5). These observations revealed that NBIF modulated immune cells in Aβ_25‐35_‐induced mice. At the same time, we found a beneficial effect of Don on the regulation of immune cells.

### 3.5 NBIF Attenuated Glial Cells Activation and Inflammation in Aβ_25‐35_‐Induced Mice

**FIGURE 6:**
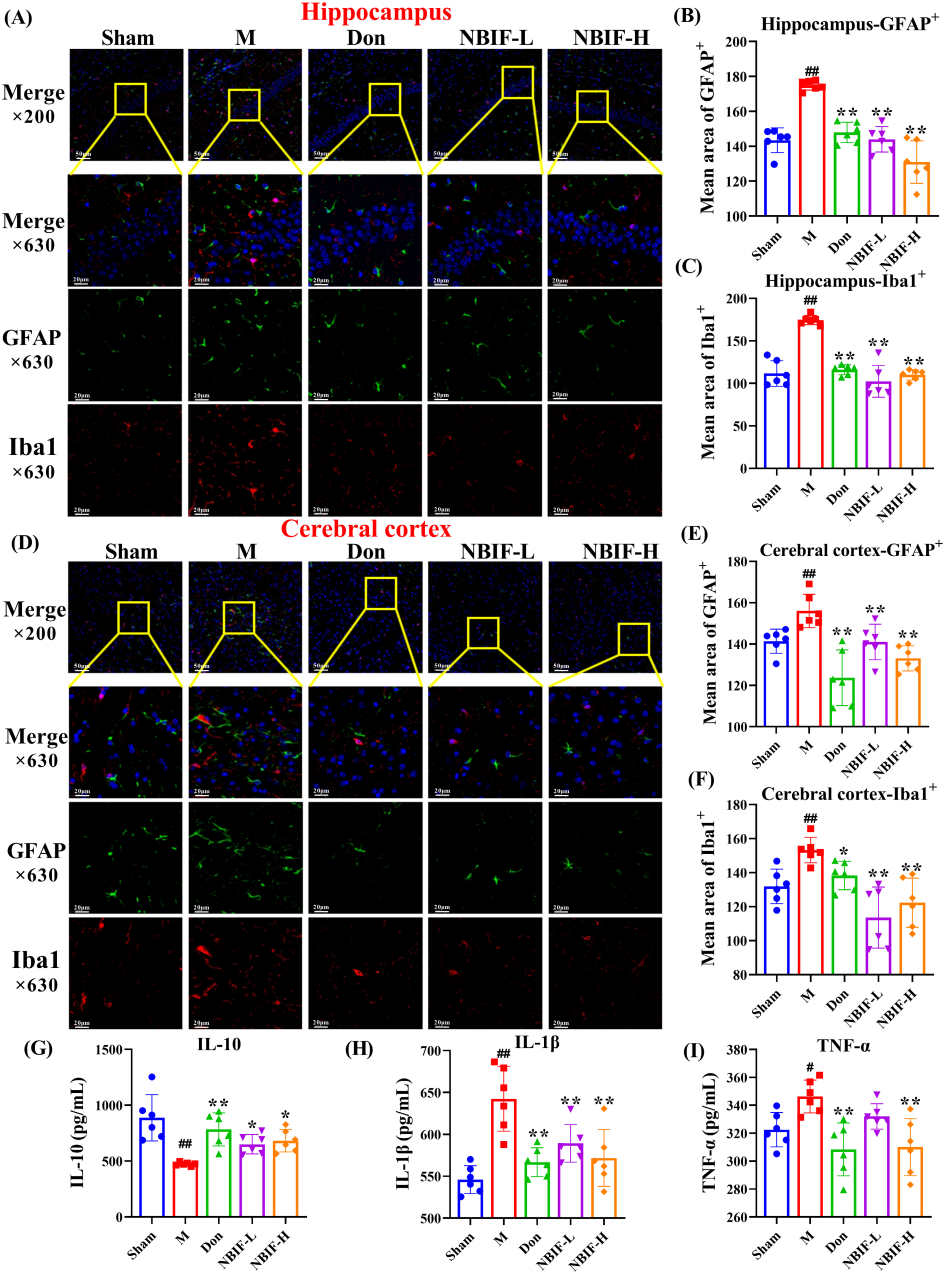
NBIF attenuated glial cells activation and inflammation in Aβ_25‐35_‐induced mice. (A–C) Representative images of glial cells in the hippocampus and quantification results of GFAP and Iba1, scale bar = 20 μm. (D–F) Representative images of glial cells in the cortical areas and quantification results of GFAP and Iba1, scale bar = 20 μm. (G–I) Analysis of IL‐10, IL‐1β, and TNF‐α in mice brain tissue. Data were expressed as the mean ± SD (*n* = 6). ^#^ *p* < 0.05 and ^##^ *p* < 0.01 versus Sham; *p < 0.05 and **p < 0.01 versus M.

Glial cells activation is closely related to neuroinflammation. To investigate the effect of NBIF on neuroinflammation, we used immunofluorescence to detect the activation levels of glial fibrillary acidic protein (GFAP) and ionized calcium binding adapter molecule 1 (Iba1), and the content of inflammatory factors in the brain tissue of Aβ_25‐35_‐induced mice. Results showed that Don and NBIF significantly downregulated GFAP and Iba1 levels in the hippocampus and cortical regions (*p* < 0.01 or *p* < 0.05, Figure 6A–F), inhibited glial cell activation, and increased the expression of IL‐10 while the expression of IL‐1β and TNF‐α decreased (*p* < 0.01 or *p* < 0.05, Figure 6G–I). These data illustrated that Don and NBIF attenuates neuroinflammation caused by glial cell activation in Aβ_25‐35_‐induced mice.

### 3.6 Drug‐Target, Disease‐Target, Target‐Pathway Network Analysis

**FIGURE 7:**
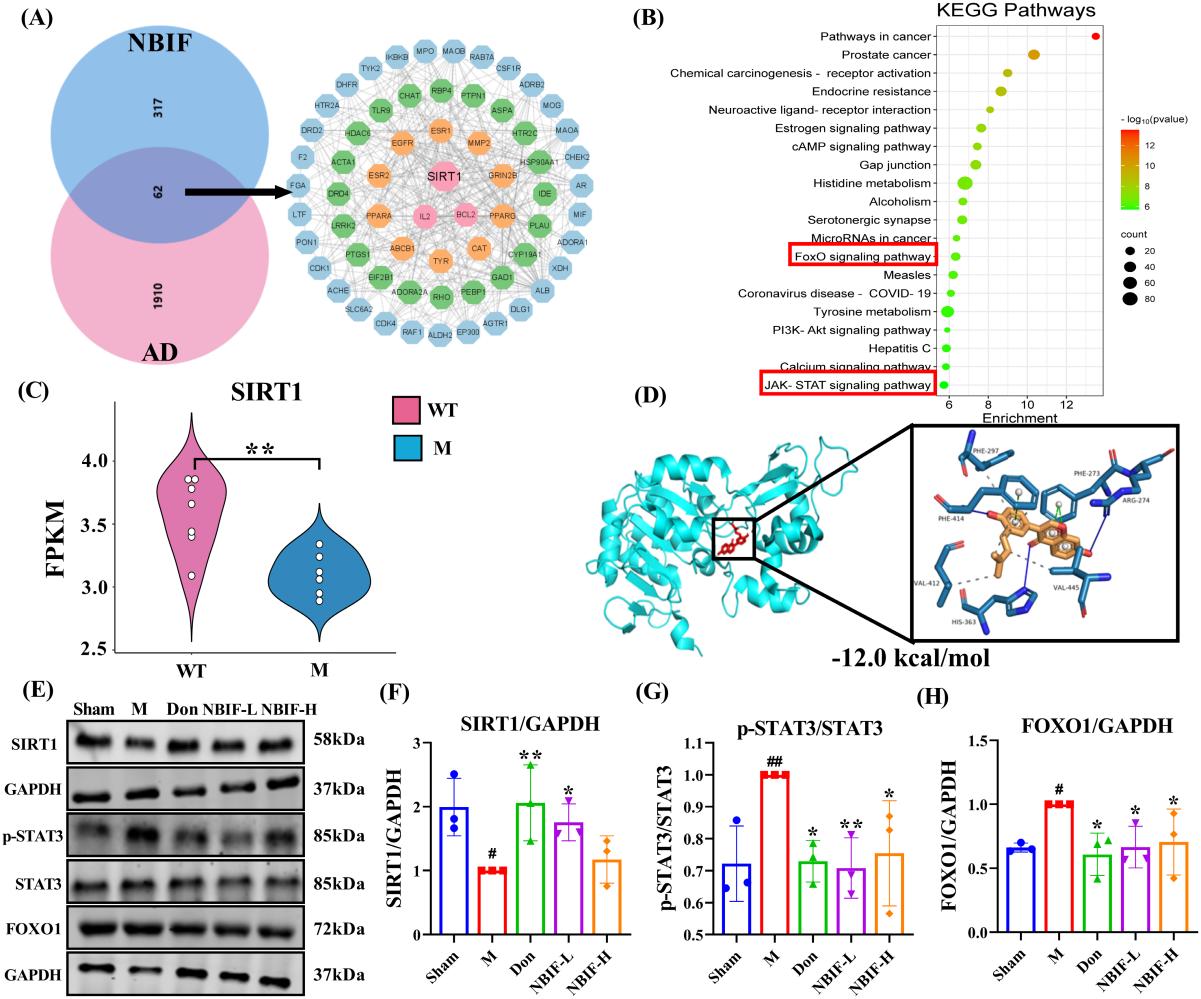
Effects of NBIF on AD based on network pharmacology. (A) Target genes of NBIF and AD. (B) Analysis of the KEGG pathway in NBIF for the treatment of AD. (C) Quantitative results of SIRT1 levels in the GEO database (*n* = 6 or 7, *p < 0.01 versus WT). (D) Molecular docking results of NBIF with SIRT1. (E–H) Representative WB images and quantitative analysis of the protein expressions of SIRT1, p‐STAT3/STAT3 and FOXO1. Data were expressed as the mean ± SD. ^#^ *p* < 0.05 and ^##^ *p* < 0.01 versus Sham; *p < 0.05 and **p < 0.01 versus M.

To further explore the mechanism of NBIF intervention in AD, we used network pharmacology to predict the targets and pathways of NBIF for the treatment of AD. The results of network pharmacology showed that NBIF treatment of AD involved 62 targets such as SIRT1 and Bcl‐2 (Figure 7A), and various signaling pathways such as neuroactive ligand‐receptor interactions, FoxO and JAK–STAT (Figure 7B). In addition, querying the GEO database revealed that SIRT1 expression was suppressed in AD mice (Figure 7C). The above results suggested that SIRT1 may play an important role in the treatment of AD by NBIF. Next, molecular simulations were performed to further investigate the molecular interactions between SIRT1 and NBIF. As shown in Figure 7D, three H‐bonds, five hydrophobic interactions and three π‐stacking occurred between NBIF and SIRT1, suggesting that NBIF treatment of AD may be associated with activation of SIRT1. Meanwhile, we examined the levels of SIRT1 and its downstream proteins STAT3 and FOXO1, and found that administration of NBIF restored the downregulation of SIRT1 and inhibited the expression of p‐STAT3 and FOXO1 in Aβ_25–35_‐induced mice (*p* < 0.01 or *p* < 0.05, Figure 7E–H). These data demonstrated that NBIF has the potential to ameliorate AD by inhibiting the STAT3/FOXO1 pathway through activation of SIRT1.

### 3.7 Silencing SIRT1 Reversed the Ameliorative Effect of NBIF on Aβ_25‐35_‐Induced N9 Cell Damage

**FIGURE 8:**
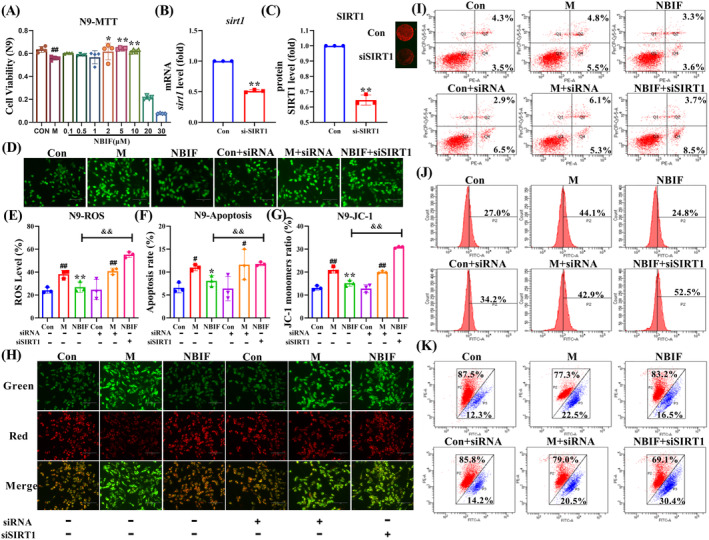
Silencing SIRT1 reversed the ameliorative effect of NBIF on Aβ25‐35‐induced N9 cells damage. (A) MTT assay for cell viability. (B and C) Effect of SIRT1 silencing on N9 cells. (D and E) Quantitative levels and representative fluorescence images of ROS in N9 cells. (F) Quantitative levels of apoptosis. (G and H) Quantification result and representative fluorescence images of JC‐1. (I–K) Representative flow cytometry images of apoptosis, ROS, and JC‐1. Data were expressed as the mean ± SD (*n* = 4 or 3). ^#^ *p* < 0.05 and ^##^ *p* < 0.01 versus Sham; *p < 0.05 and **p < 0.01 versus M; ^&&^ *p* < 0.01 versus NBIF‐siSIRT1.

To further investigate the role of NBIF through SIRT1, Aβ_25‐35_ was used to induce N9 cell injury, and apoptosis, ROS and JC‐1 metrics were measured. MTT results showed that both 2 μM and above NBIF significantly increased the cell viability of Aβ_25‐35_‐induced N9 cells, and the effect was better at 5 μM (*p* < 0.01, Figure 8A). Next, siSIRT1 is used to detect if NBIF is functioning through SIRT1. As shown in Figure 8B,C, the levels of *Sirt1* mRNA and SIRT1 protein in the siSIRT1 group were extremely significantly lower than those of the Con group (*p* < 0.01). This suggested that siSIRT1 inhibits *Sirt1* mRNA and SIRT1 protein in N9 cells. Figure 8D–K showed that Aβ_25‐35_ treatment significantly increased apoptosis, ROS and JC‐1 levels in N9 cells (*p* < 0.01 or *p* < 0.05). Compared with the M group, NBIF significantly adjusted the levels of apoptosis, ROS and JC‐1 (*p* < 0.01 or *p* < 0.05). However, the presence of siSIRT1 inhibited these effects of NBIF. This suggested that NBIF may attenuate the cellular damage caused by Aβ_25‐35_ through SIRT1 target.

### 3.8 Effect of NBIF on the SIRT1 Signaling Pathway in N2a‐APP695 Cells

**FIGURE 9:**
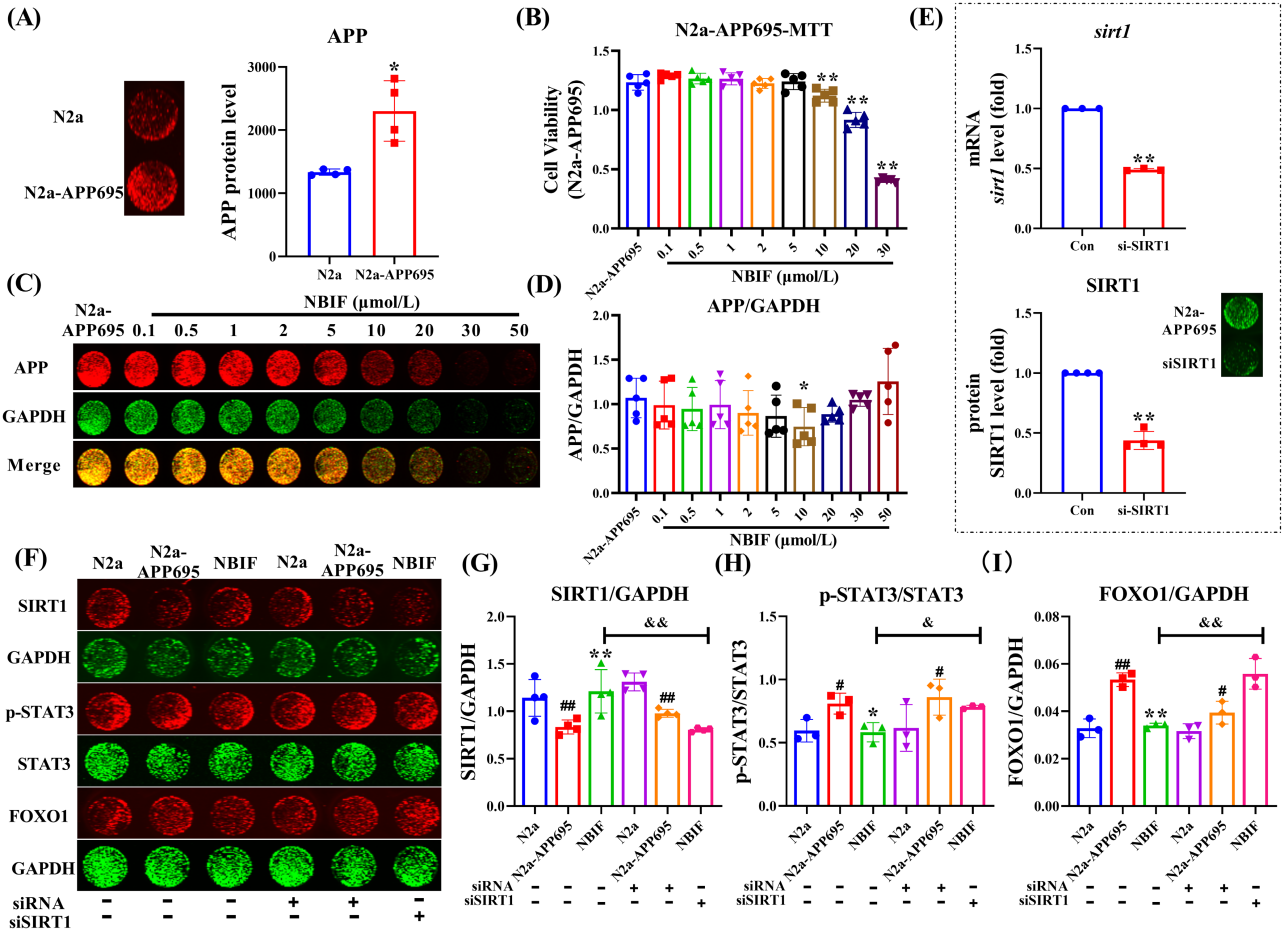
Effect of NBIF on the SIRT1‐STAT3/FOXO1 signaling pathway in N2a‐APP695 cells. (A) Results of ICW detection of APP expression in N2a and N2a‐APP695 cells. (B) MTT assay for cell viability. (C and D) Effects of different concentrations of NBIF on APP protein expression in N2a‐APP695 cells. (E) Effect of SIRT1 silencing on N2a‐APP695 cells. (F–I) Effect of NBIF on SIRT1‐STAT3/FOXO1 pathway‐related proteins. Data were expressed as the mean ± SD (*n* = 3–5). ^#^ *p* < 0.05 and ^##^ *p* < 0.01 versus Sham; *p < 0.05 and **p < 0.01 versus M; ^&^ *p* < 0.05 and ^&&^ *p* < 0.01 versus NBIF‐siSIRT1.

In order to further explore the effect of NBIF on the SIRT1 signaling pathway, we used N2a‐APP695 cells. As shown in Figure 9A, the levels of APP were significantly increased in the N2a‐APP695 cells compared to the N2a cells (*p* < 0.05). At 10 μM and above, NBIF significantly inhibited the abnormal proliferation of N2a‐APP695 cells (*p* < 0.01, Figure 9B). And ICW results showed that NBIF significantly inhibited APP expression at 10 μM (*p* < 0.05, Figure 9C,D). Next, siSIRT1 was used to detect the effect of NBIF on SIRT1 pathway‐related proteins. As shown in Figure 9E, siSIRT1 significantly reduced Sirt1 mRNA and SIRT1 protein levels in N2a‐APP695 cells (*p* < 0.01). The ICW results showed that the protein expression level of SIRT1 was significantly reduced in the M group compared to the control group, and level of p‐STAT3/STAT3 and FOXO1 in the M group were higher than the control group (*p* < 0.05 or *p* < 0.01, Figure 9F–I). Nevertheless, siSIRT1 treatment differentially reversed the expression levels of these proteins.

## 4 Discussion

AD is the most common form of dementia [21], which is characterized by memory loss, cognitive impairment, and a deterioration in daily activities [22]. Donepezil, an acetylcholinesterase inhibitor, is commonly used in the treatment of AD [17], so donepezil was chosen as a positive control in this experiment. Behavioral indicators such as open field test, novel object recognition test, and morris water maze test can be used to assess the cognitive and learning memory abilities of animals through changes in animal behavior [23]. In addition, neuronal damage occurs during the development of AD. The level of apoptosis and the number of neurons reflect the neuronal damage [24]. This study showed that Don and NBIF significantly ameliorated cognitive deficits and memory deficits in Aβ_25‐35_‐injected mice, as well as effectively attenuated neuronal loss and levels of apoptotic cells in hippocampal tissue.

Although the pathogenesis of AD is not completely clear, the amyloid cascade hypothesis remains one of the most recognized mechanisms for understanding AD [25]. Amyloid β‐protein (Aβ) is the end product of several enzymes breaking down amyloid precursor protein (APP). Under normal conditions, α‐secretase and γ‐secretase hydrolyzes APP, preventing the production of Aβ. Yet once the disease occurs, APP is hydrolyzed by the β‐site APP cleavage enzyme 1 (BACE1) and γ‐secretase to produce toxic Aβ fragments [26], the most common of which are Aβ40 and Aβ42, which make up the majority of Aβ plaques [27]. During the development of AD, there is competition for APP cleavage between α‐secretase and BACE1 in the same protein hydrolysis area, and BACE1 activity increases while α‐secretase cleavage decreases [28]. It has been suggested that a disintegrin and metalloprotease 10 (ADAM10) is the major α‐secretase [29] and that increasing ADAM10 expression reduces Aβ production. Thus, blocking the formation of toxic Aβ plaques by affecting enzyme activity could be a therapeutic option for AD [30]. Our results revealed that after Don and NBIF treatment, BACE1 protein levels were reduced while ADAM10 levels were increased, and Aβ levels were reduced in mouse brain tissue. This suggests that Don and NBIF may restore the balance of APP hydrolysis and reduce toxic Aβ levels by increasing α‐secretase activity through inhibiting β‐secretase expression.

The neuroinflammatory response is also a key factor in the progression of AD. Activated microglia, reactive astrocytes and immune cell infiltration are major factors in neuroinflammation [31]. Microglia and astrocytes are the main cells involved in innate immune cells [32]. When amyloid plaques build in the brain, microglia detect them and become activated, triggering an inflammatory response and the production of a significant number of pro‐inflammatory cytokine molecules, such as IL‐6, IL‐1β, and TNF‐α [33]. Nevertheless, overactivation of microglia reduces their ability to phagocytose Aβ and also increases the ability of astrocytes to transform into reactive astrocytes [34]. The synergistic reaction between reactive astrocytes and activated microglia not only leads to a sustained release of inflammatory factors, but also exacerbates the accumulation of Aβ and tau, promotes oxidative stress, and accelerates neuronal death, all of which contribute to the progression of AD [35]. Furthermore, the pro‐inflammatory environment activates the adaptive immune system, resulting in the recruitment of peripheral immune cells into the brain tissue via the damaged blood–brain barrier [36]. After intervention with NBIF, the activation levels of glial cells were suppressed in mice hippocampal and cortical, and the levels of MDSCs and Tregs in the blood were differentially downregulated, and the levels of NKs and Thcs were upregulated. We speculate that NBIF ameliorates Aβ_25–35_‐induced neuroinflammation in mice by inhibiting glial cell activation and modulating immune cells.

Network pharmacology results confirm that SIRT1 and STAT3/FOXO1 are key targets and pathways for NBIF in the treatment of AD. Sirtuin 1 (SIRT1) is a histone deacetylase that plays an essential role in delaying cellular senescence [37], regulating mitochondrial function, apoptosis, and inflammation [38]. Studies have shown that SIRT1 upregulates α‐secretase [39] and inhibits β‐secretase activity to reduce toxic Aβ production [40] and improve AD. Results from the GEO database further confirm the important role of SIRT1 in AD. Reduced SIRT1 levels activate signal transducer and activator of transcription 3 (STAT3) [41], and abnormal STAT3 activation enhances neuroinflammation‐related cytokine production [42], exacerbating AD. Forkhead box protein O1 (FOXO1), a downstream target of STAT3 [43], regulates physiological processes such as cell proliferation, apoptosis, and antioxidative stress. Recent research has revealed that FOXO1 is intimately associated with neurological illnesses, and that activation of FOXO proteins causes cell death [44]. We evaluated associated protein expression and discovered that NBIF increased SIRT1, blocked STAT3 activation, and lowered FOXO1 protein expression. Moreover, we conducted in vitro tests using gene silencing to verify whether NBIF improved AD via the SIRT1‐mediated STAT3/FOXO1 pathway. Transfection silencing of SIRT1 abrogated the beneficial effect of NBIF on Aβ_25‐35_‐induced N9 damage and associated protein expression in N2a‐APP695 cells. These data suggest that NBIF may ameliorate neuroinflammation‐mediated AD by modulating the SIRT1‐mediated STAT3/FOXO1 pathway.

In conclusion, NBIF enhances learning and memory abilities in Aβ_25‐35_‐induced mice and ameliorates brain injury by regulating apoptosis, oxidative stress, and neuroinflammation, by a mechanism that may be related to activation of SIRT1. Silencing of SIRT1 by transfection further confirmed the critical role of SIRT1 in N2a‐APP695 cells and Aβ_25‐35_‐induced N9 cells. This study implies that NBIF may be a potential medicine for the treatment of AD, and the findings of this study have important significance for AD drug development.

## Author Contributions

Fengxiao Hao, Mengnan Zeng, and Xiaoke Zheng contributed to the experimental design and writing of the manuscript. Bing Cao, Xiwen Liang, Xinmian Jiao, and Kaili Ye performed the experiments and analyzed the data. Weisheng Feng, Xiaoke Zheng, and Mengnan Zeng supervised the project. All authors read and agree to publish the final manuscript.

## Ethics Statement

All animal experiments were approved by the ethics committee of Henan University of Chinese Medicine and performed under the institutional guidelines. The Ethical Committee approved the animal procedure of the Henan University of Chinese Medicine (DWLL2018080003).

## Conflicts of Interest

The authors declare no conflicts of interest.

## Supporting information


Appendix S1.

## Data Availability

The data that support the findings of this study are available from the corresponding author upon reasonable request.
