# Supplementary material for: Neobavaisoflavone Ameliorates Memory Deficits and Brain Damage in Aβ25‐35‐Induced Mice by Regulating SIRT1
Source: CNS Neurosci Ther. 2024 Oct 11;30(10):e70068. doi: 10.1111/cns.70068 (PMC11469773; doi:10.1111/cns.70068)

Full unedited gel/blot for Figure 3H

ADAM10

84 kDa→

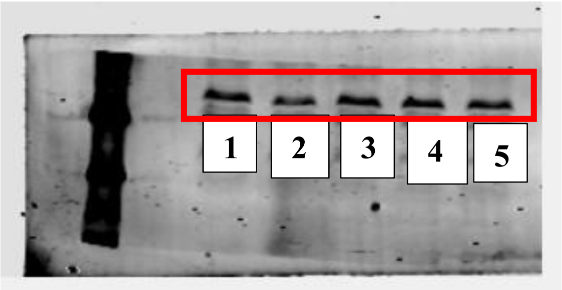

1: Sham; 2: M; 3: Don;4: NBIF-L (15 mg/kg); 5: NBIF-H (30 mg/kg)

GAPDH

37 kDa→

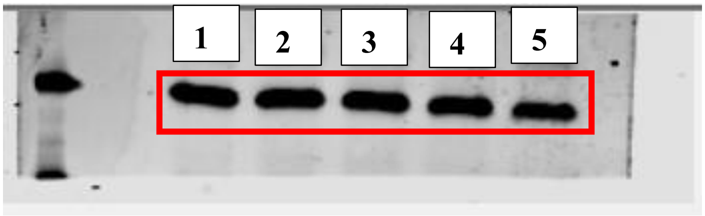

BACE1

70 kDa→

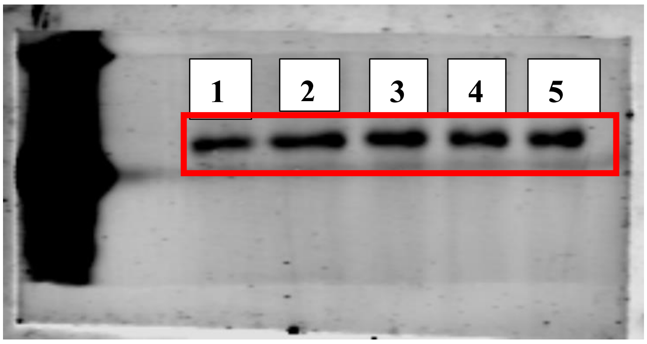

GAPDH

37 kDa→

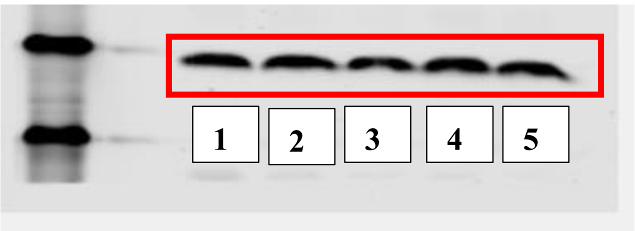

Full unedited gel/blot for Figure 4D

Bcl-2

26 kDa→

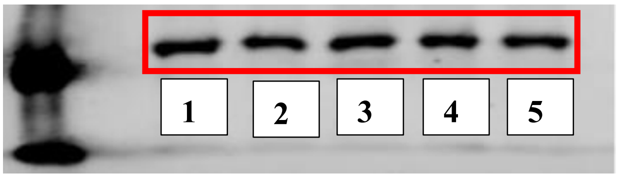

1: Sham; 2: M; 3: Don;4: NBIF-L (15 mg/kg); 5: NBIF-H (30 mg/kg)

Bax

20 kDa→

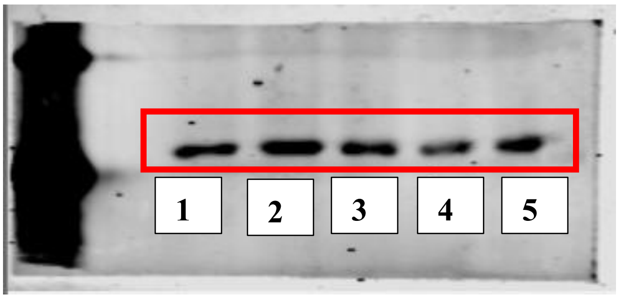

Caspase-3

34 kDa→

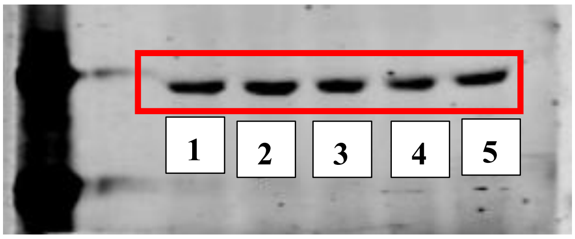

Caspase-9

46 kDa→

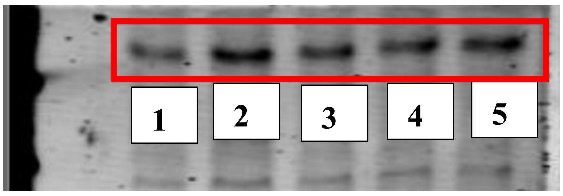

β-Tubulin

55 kDa→

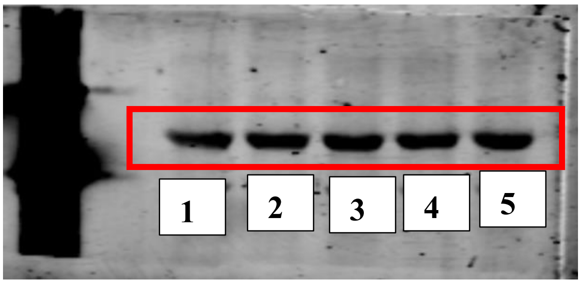

Full unedited gel/blot for Figure 7E

SIRT1

58 kDa→

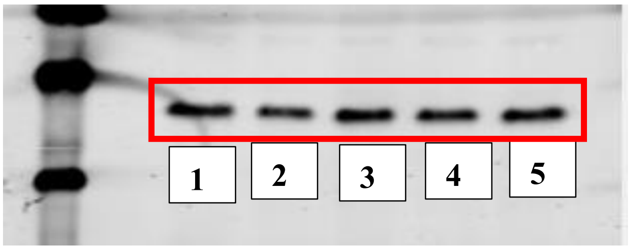

1: Sham; 2: M; 3: Don;4: NBIF-L (15 mg/kg); 5: NBIF-H (30 mg/kg)

GAPDH

37 kDa→

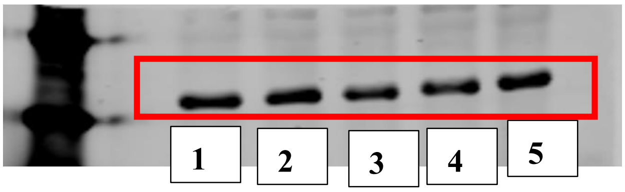

p-STAT3

85 kDa→

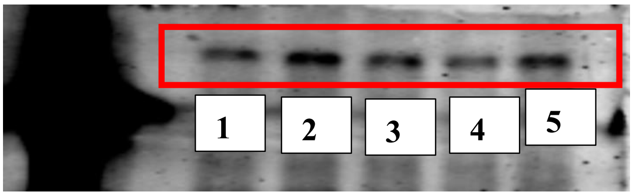

STAT3

85 kDa→

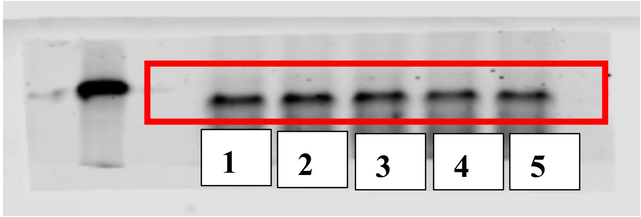

FOXO1

72 kDa→

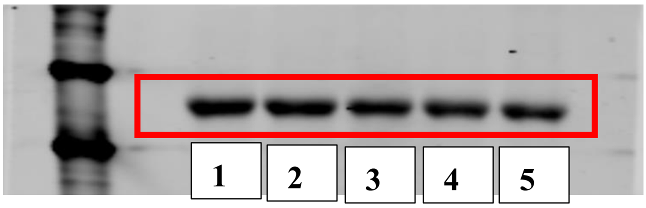

GAPDH

37 kDa →

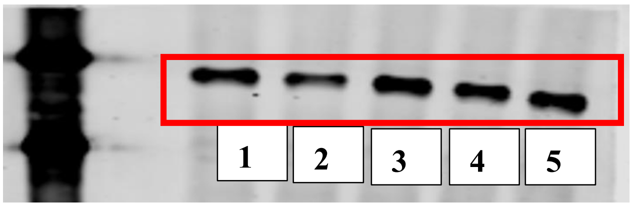

Supplement: Supplementary file 1 — Appendix S1. [file CNS-30-e70068-s001.pdf]
